# Inhibitory effects of sulfur dioxide within the nucleus tractus solitarii of rats: involvement of Calcium Ion channels, Adenine nucleoside triphosphate-sensitive potassium channels, and the nitric oxide/cyclic Guanine trinucleotide phosphate pathway

**DOI:** 10.1097/WNR.0000000000001304

**Published:** 2019-08-15

**Authors:** Bin Li, Ming-Xia Gao, Wei-lin Yang, Chen Chai, Deng-xia Zhang, Hong-Yan Cai, Jian Liu, Yan Lu

**Affiliations:** a Department of General Surgery; b Department of Reproductive Medicine, Maternity Hospital, First Hospital of Lanzhou University, Lanzhou; c Department of Gynaecology and Obstetrics, Changzhou Second People’s Hospital, Nanjing Medical University, Changzhou; d Department of Intensive Care Unit, Second Hospital of Lanzhou University; e Department of Intensive Care Unit, First Hospital of Lanzhou University, Lanzhou; f Department of Clinical Laboratory, San Ai Tang Hospital, Lanzhou, China

**Keywords:** baroreflex sensitivity, cyclic GMP, nitric oxide, nucleus tractus solitarii, sulfur dioxide

## Abstract

This study was designed to investigate the cardiovascular effects of sulfur dioxide within the nucleus tractus solitarii. Sulfur dioxide or artificial cerebrospinal fluid was unilaterally applied into the nucleus tractus solitarii of rats, and the effects on blood pressure, heart rate, and arterial baroreflex sensitivity (ABR) were determined. To explore the mechanisms of the effects of intra-nucleus tractus solitarii sulfur dioxide, various inhibitors were applied prior to sulfur dioxide treatment. Unilateral microinjection of sulfur dioxide produced a dose-dependent decrease in blood pressure in anesthetized rats. Significant decreases in heart rate were also seen after unilateral microinjection of 20 and 200 pmol of sulfur dioxide (*P* < 0.05). Bilateral microinjection of sulfur dioxide into the nucleus tractus solitarii significantly decreased blood pressure and heart rate and also attenuated ABR. Pretreatment with glibenclamide or nicardipine within the nucleus tractus solitarii did not alter the hypotension or bradycardia (*P* > 0.05) induced by intra-nucleus tractus solitarii sulfur dioxide. Pretreatment with 1H-[1,2,4]Oxadiazolo[4,3-a]quinoxalin-1-one, however, significantly attenuated this hypotension and bradycardia. Prior application of kynurenic acid or *N*(G)-Nitro-l-arginine methyl ester into the nucleus tractus solitarii partially diminished the hypotension and bradycardia induced by intra-nucleus tractus solitarii sulfur dioxide. Our present study shows that sulfur dioxide produces cardiovascular inhibitory effects in the nucleus tractus solitarii, predominantly mediated by glutamate receptors and the nitric oxide/cyclic GMP signal transduction pathway.

## Introduction

Sulfur dioxide (SO_2_) is a major toxic gas and environmental pollutant [1]. Its toxicity has been extensively investigated in humans, animals, and plants [2]. SO_2_, however, is endogenously produced during the metabolism of sulfur-containing amino acids in mammals [3]. It has also been shown to exert extensive physiological and pathological functions in cardiovascular system [3]. With a relative molecular mass of 64 g/mol, SO_2_ can readily pass through the membrane independently of a specific receptor. On the basis of these studies, SO_2_ has been proposed as a novel endogenous gaseous signaling molecule alongside nitric oxide (NO), carbon monoxide, and hydrogen sulfide [4]. We have previously shown that aspartate aminotransferase (AAT), a key enzyme in endogenous SO_2_ generation, is produced in the mammalian brain [5] and causes hypertension and tachycardia [6]. The central nervous system (CNS) is known to be involved in regulating arterial pressure [7–9]. The present study was designed to test the hypothesis that SO_2_ regulates blood pressure (BP) by a central mechanism.

It is well known that the nucleus tractus solitarii (NTS) has a key role in regulating BP [10]. Neurons of the nucleus tractus solitarii (NTS) project directly or indirectly to structures of the medulla that regulate the activity of sympathetic and parasympathetic preganglionic neurons [11]. Although the NTS plays a key role in mediating tonic and reflex control of the cardiovascular system, the cardiovascular effects of SO_2_ in the NTS have not been determined. Accordingly, the present study was designed to investigate the cardiovascular effects of intra-NTS SO_2_.

## Materials and methods

### Animals

Experiments were performed on 78 adult male Sprague–Dawley rats (2.5–3.0 kg) obtained from the Lanzhou University Laboratory Animal Center. Rats were housed under standard laboratory conditions, with a 12/12-h light/dark cycle and controlled temperature (23 ± 2°C). Animal treatment complied with the National Institutes of Health (NIH Publication 80-23) and our institute guidelines for the care and use of laboratory animals. Animal preparation, microinjection, and histological procedures were performed as described previously [12].

### General procedures

Briefly, after 3 days of acclimatization, animals were anesthetized with pentobarbital sodium (40 mg/kg, intraperitoneal injection (i.p.)) or combined anesthesia (800 mg/kg urethane, 40 mg/kg α-chloralose, and 40 mg/kg sodium tetraborate; i.p.). A trachea was then cannulated and connected to an animal ventilator (DW-2000; Shanghai Jiapeng Technology Co. Ltd, Shanghai, China) to facilitate mechanical respiration (10–12 ml/kg, 60–70 times/min). Except for those being measured for baroreflex sensitivity, rats were paralyzed with gallamine triethiodide (10 mg/kg initially and then 4 mg/kg every 30 min, intravenous injection (i.v.)) and artificially ventilated with oxygen-enriched room air. The right femoral artery and vein were then cannulated with polyethylene catheters to measure arterial BP and administer drugs, respectively. Mean arterial pressure (MAP) and heart rate (HR) were calculated using an RM6240 recording system (Chengdu Technology Co. Ltd, Chengdu, China). When necessary, supplemental doses of α-chloralose (20 mg/kg, i.v.) were administered to maintain an appropriate level of anesthesia. Subsequently, rats were fixed in a stereotaxic apparatus (MP-8003; RWD life Technology Co. Ltd, Shenzhen, China), and the dorsal surface of the medulla was surgically exposed. Body temperature was maintained at approximately 37°C with an infrared heating lamp.

### Microinjection procedure

Coordinates for microinjections into the NTS were determined by stereotaxic atlas. A multi-barreled micropipette (tip diameter 20–30 μm) was inserted into the NTS (0.5–0.8 mm rostral to the obex, 0.5–1.0 mm lateral to the midline, and 0.2–0.5 mm below the dorsal surface of the medulla). The micropipette was filled with l-glutamate, SO_2_ (2, 20, or 200 pmol), glibenclamide, nicardipine, N(G)-Nitro-l-arginine methyl ester (l-NAME), 1H-[1,2,4]Oxadiazolo[4,3-a] quinoxalin-1-one (ODQ), kynurenic acid (KYN), or artificial cerebrospinal fluid (aCSF) using a microsyringe. Glibenclamide, nicardipine, and ODQ were initially dissolved in dimethyl sulfoxide (DMSO) and then diluted to the final concentration with aCSF (133.3 mM sodium chloride, 3.4 mM potassium chloride, 1.3 mM calcium chloride, 1.2 mM Magnesium chloride, 0.6 mM Sodium dihydrogen phosphate, 32.0 mM Sodium bicarbonate, and 3.4 mM glucose). pH was adjusted to 7.4 with 10% HCl. The final concentration of DMSO in aCSF was less than 1%, which produced little effect on BP or HR of rats in preliminary experiments. Other drugs were dissolved directly in aCSF. The selected drugs were based on preliminary experiments and previous study [12]. Drugs were prepared over a period of 5 to 10 s, and the injection volume (100 nl) was carefully measured by observing the movement of the fluid meniscus along a reticule under the guidance of an operating microscope. Each animal received only one microinjection into the NTS. Functional identification of the NTS was based on obtaining a depressor response to a microinjection of glutamate (2 nmol).

### Determination of baroreflex sensitivity

After surgery, rats were stabilized for at least 30 min before baseline BP and HR measurements were taken. Baroreflex sensitivity was measured in rats given combined anesthesia, using a method described previously [12]. In brief, a bolus intravenous injection of phenylephrine (10 μg/kg) was administered to raise systolic BP (SBP) by 20 to 40 mmHg, before and 5 or 30 min after, bilaterally injecting SO_2_ into the NTS (20 pmol each side). At least 10 min was allowed between phenylephrine injections. The relationship between heart beat period and SBP was analyzed by linear regression analysis (correlation coefficient, *R*^2^ > 0.8) to determine ABR (arterial baroreflex reflex, heart beat period [ms]/SBP [mmHg]).

### Histological analysis

After completing experiments, an overdose of urethane (0.4 g/kg, i.v.) was applied to deeply anesthetize the rats and 20 μl of 2% pontamine sky blue solution was injected to verify the microinjection sites. The brain stem was then removed and fixed for 48 to 72 h in a 10% paraformaldehyde–saline solution containing 30% sucrose. Subsequently, frozen brains were sectioned (50 μm) in the coronal view and stained with neutral red.

### Statistical analysis

All values were expressed as mean ± SEM. BP is presented as MAP, calculated from the following formula: diastolic BP + [(systolic − diastolic BP)/3]. MAP and HR before and after treatment were analyzed by repetitive-measure analysis of variance (ANOVA). Multiple groups’ means were compared by one-way ANOVA and Newman–Keuls test. A value of *P* < 0.05 was considered statistically significant.

## Results

### Cardiovascular responses to microinjection of sulfur dioxide into the nucleus tractus solitarii

**Fig. 1:**
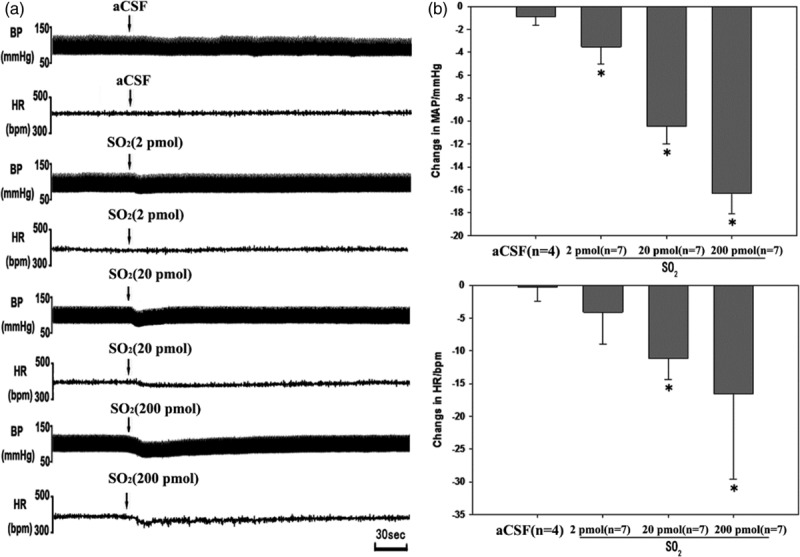
Topical application of SO_2_-induced hypotension and bradycardia. (a) Representative original tracings showing the BP and HR response by unilateral microinjection of SO_2_ (2–200 pmol) or aCSF (100 nl) into the NTS of rats; (b) Magnitude of changes in MAP and HR by unilateral microinjection of SO_2_ (2–200 pmol) or aCSF (100 nl) into the NTS (mean ± SEM). *P < 0.05 vs vehicle (aCSF). aCSF, artificial cerebrospinal fluid; BP, blood pressure; HR, heart rate; MAP, mean arterial pressure; NTS, nucleus tractus solitarii; SO_2_, aqueous solution of sulfur dioxide.

Figure 1a shows representative traces of BP and HR responses to the microinjection of SO_2_ (2–200 pmol) or aCSF (100 nl) into the NTS. Intra-NTS injection of aCSF did not alter basal MAP [106 ± 16 vs 105 ± 16 mmHg, *F* (1, 3) = 5.703, *P* > 0.05] or HR [438 ± 23 vs 438 ± 24 bpm, *F* (1, 3) = 0.000, *P* > 0.05]. Topical application of SO_2_, however, produced dose-dependent hypotension (2 pmol: −4 ± 1 mmHg; 20 pmol: −10 ± 2 mmHg; 200 pmol: −16 ± 2 mmHg) in anesthetized rats [*F* (1, 21) = 635.936, *P* < 0.05, compared with microinjection of aCSF: −1 ± 1 mmHg]. Although microinjection of a low dose of SO_2_ (2 pmol) into the NTS did not significantly influence HR [−4 ± 5 bpm; *F* (1, 9) = 2.475, *P* > 0.05, compared with microinjection of aCSF: −0 ± 2 bpm], microinjection of higher doses (20 and 200 pmol) produced significant bradycardia [20 pmol: −11 ± 3 bpm; 200 pmol: −17 ± 13 bpm vs aCSF: 0 ± 3 bpm; *F* (1, 15) = 19.506, *P* < 0.05]. Hypotension and bradycardia occurred 5 s after topical application of SO_2_, reached their nadir after 20 s, and returned to baseline levels after approximately 2 min. The cardiovascular responses to microinjection of aCSF and SO_2_ are summarized in Fig. 1b.

### Effects of intra-nucleus tractus solitarii sulfur dioxide microinjections on ABR

**Fig. 2:**
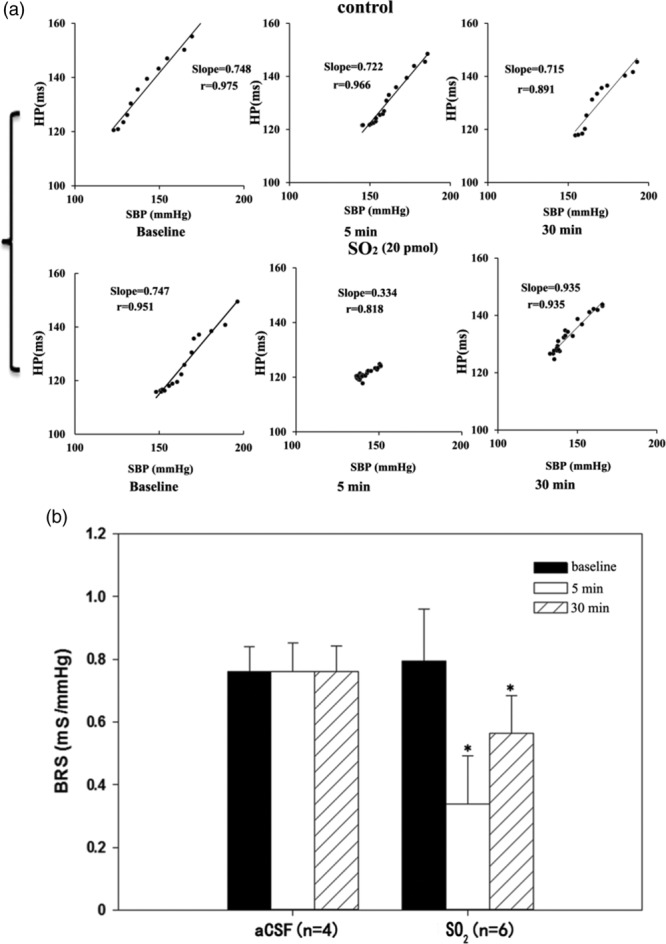
The effects of bilateral microinjection of SO_2_ on BP and HR responses induced by phenylephrine. (a) The sample traces of phenylephrine-evoked baroreflex before and after 5 and 30 min of microinjection of SO_2_ (20 pmol for each side, n = 6) or vehicle (aCSF, 100 nl for each side, n = 4) into the NTS. Values of slope are the values of baroreflex sensitivity. (b) Responses of BRS before and after 5 min, 30 min of microinjection of SO_2_ (20 pmol), or aCSF(100 nl) into the NTS. aCSF, artificial cerebrospinal fluid; BP, blood pressure; BRS, baroreflex sensitivity; HR, heart rate; HP, heart beat period; NTS, nucleus tractus solitarii; SBP, systolic blood pressure; SO_2_, aqueous solution of sulfur dioxide.

Fig. 2a and b shows the effects of the phenylephrine-evoked baroreflex before, 5 min after, and 30 min after bilateral microinjection of SO_2_ into the NTS. Bilateral microinjection of the vehicle, aCSF, did not alter basal ABR [5 min: 0.762 ± 0.091 ms/mmHg; 30 min: 0.760 ± 0.083 ms/mmHg vs control: 0.761 ± 0.078 ms/mmHg; *F* (2, 6) = 0.033, *P* > 0.05]. Bilateral microinjection of SO_2_ into the NTS, however, significantly decreased basal MAP [from 105 ± 12 to 93 ± 12 mmHg, *F* (1, 6) = 336.940, *P* < 0.05] and HR [from 430 ± 25 to 416 ± 27 bpm, *F* (1, 6) = 82.964, *P* < 0.05] and attenuated ABR [5 min: 0.338 ± 0.154 ms/mmHg; 30 min: 0.564 ± 0.120 ms/mmHg vs control: 0.795 ± 0.166 ms/mmHg; *F* (2, 18) = 89.141, *P* < 0.05]. The effects of intra-NTS microinjection of SO_2_ and aCSF on ABR are summarized in Fig. 2.

### Effects of ATP-sensitive potassium blockade, l-type calcium channel blockade, and soluble guanylyl cyclase inhibition on cardiovascular functions affected by intra-nucleus tractus solitarii sulfur dioxide

Table 1 summarized the BP and HR responses to intra-NTS SO_2_ following pretreatment with the ATP-sensitive potassium (K_ATP_) blocker, glibenclamide, the l-type calcium channel blocker, nicardipine, and the soluble guanylyl cyclase (sGC) inhibitor, ODQ. In vehicle, prior microinjection of the vehicle, consisting of aCSF in 1% DMSO, did not alter basal MAP [108 ± 4 vs 109 ± 5 mmHg, *F* (1, 3) = 0.045, *P* > 0.05] or HR [413 ± 42 vs 414 ± 42 bpm, *F* (1, 3) = 0.033, *P* > 0.05] and did not affect the hypotension and bradycardia induced by intra-NTS SO_2_. Microinjection of glibenclamide into the NTS transiently decreased basal MAP [from 107 ± 5 to 103 ± 5 mmHg, *F* (1, 3) = 67.079, *P* < 0.05], but did not significantly alter basal HR [from 403 ± 27 to 398 ± 27, *F* (1, 6) = 3.102, *P* > 0.05]. Pretreatment with glibenclamide did not affect the hypotensive [−9 ± 2 mmHg pretreatment with glibenclamide vs −10 ± 1 mmHg pretreatment with aCSF, *F* (1, 9) = 0.105, *P* > 0.05] or bradycardic [−10 ± 2 bpm pretreatment with glibenclamide vs −13 ± 6 bpm pretreatment with aCSF, *F* (1, 9) = 0.133, *P* > 0.05] responses to SO_2_ within the NTS. Microinjection of nicardipine into the NTS transiently decreased basal MAP [from 103 ± 11 to 97 ± 10 mmHg, *F* (1, 6) = 30.048, *P* < 0.05] and basal HR [from 411 ± 39 to 404 ± 39, *F* (1, 6) = 0.072, *P* < 0.05]. Pretreatment with nicardipine, however, did not affect the hypotensive [−10 ± 3 mmHg pretreatment with nicardipine vs −10 ± 1 mmHg pretreatment with aCSF, *F* (1, 9) = 0.700, *P* > 0.05] or bradycardic [−11 ± 6 bpm pretreatment with nicardipine vs −13 ± 6 bpm pretreatment with aCSF, *F* (1, 9) = 0.001, *P* > 0.05] responses to SO_2_. Microinjection of ODQ into the NTS significantly decreased basal MAP [from 103 ± 11 to 90 ± 10 mmHg, *F* (1, 6) = 35.993, *P* < 0.05] and basal HR [from 413 ± 46 to 398 ± 46, *F* (1, 6) = 11.024, *P* < 0.05]. It also decreased the hypotension [−3 ± 1 mmHg pretreatment with ODQ vs −10 ± 1 mmHg pretreatment with aCSF, *F* (1, 9) = 96.814, *P* < 0.05] and bradycardia [−3 ± 3 bpm pretreatment with ODQ vs −13 ± 6 bpm pretreatment with aCSF, *F* (1, 9) = 14.431, *P* < 0.05] induced by intra-NTS SO_2_. These results are summarized in Fig. 1 and Table 1.

**Table 1:**
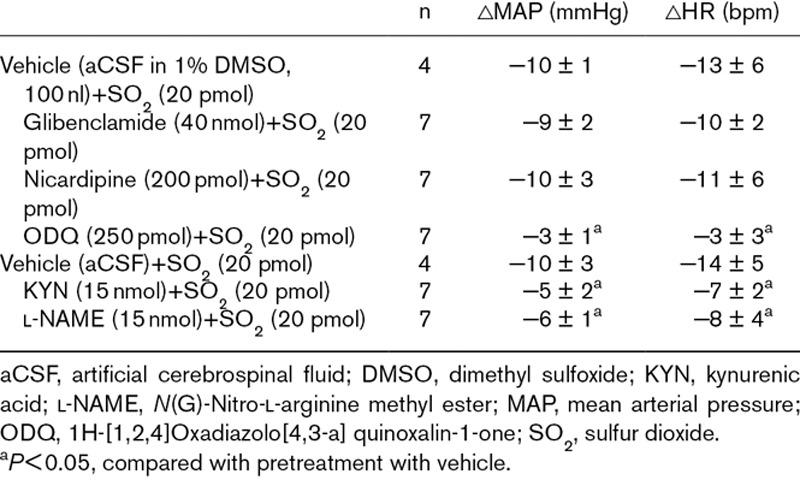
The blood pressure and heart rate responses to intra-nucleus tractus solitarii sulfur dioxide following pretreatment with the following inhibitors

### Effects of glutamate receptor antagonism and nitric oxide synthase inhibition on the cardiovascular functions affected by intra-nucleus tractus solitarii sulfur dioxide

Table 1 summarized BP and HR responses to intra-NTS SO_2_ following pretreatment with the glutamate receptor antagonist, KYN, and the non-specific NO synthase inhibitor, l-NAME. Prior microinjection of aCSF did not alter basal MAP [110 ± 7 vs 109 ± 8 mmHg, *F* (1, 3) = 0.186, *P* > 0.05] or HR [407 ± 32 vs. 406 ± 42 bpm, *F* (1, 3) = 0.125, *P* > 0.05] and did not affect the hypotension and bradycardia induced by intra-NTS SO_2_. Microinjection of KYN into the NTS significantly increased basal MAP [from 109 ± 8 to 115 ± 9 mmHg, *F* (1, 6) = 26.528, *P* < 0.05] and basal HR [from 415 ± 44 to 422 ± 43, *F* (1, 6) = 8.132, *P* < 0.05] and significantly decreased the hypotensive [−5 ± 2 mmHg pretreatment with KYN vs −10 ± 3 mmHg pretreatment with aCSF, *F* (1, 9) = 12.814, *P* < 0.05] and bradycardic [−7 ± 2 bpm pretreatment with KYN vs −14 ± 5 bpm pretreatment with aCSF, *F* (1, 9) = 7.998, *P* < 0.05] responses of SO_2_ to intra-NTS SO_2_. Microinjection of l-NAME into the NTS significantly decreased basal MAP [from 102 ± 10 to 92 ± 10 mmHg, *F* (1, 6) =65.006, *P* < 0.05] and basal HR [from 429 ± 52 to 416 ± 53, *F* (1, 6) = 41.041, *P* < 0.05] and significantly decreased the hypotension [−6 ± 1 mmHg pretreatment with l-NAME vs −10 ± 3 mmHg pretreatment with aCSF, *F* (1, 9) =16.716, *P* < 0.05] and bradycardia [−8 ± 4 bpm pretreatment with l-NAME vs −14 ± 5 bpm pretreatment with aCSF, *F* (1, 9) =11.755, *P* < 0.05] induced by intra-NTS SO_2_. These results are summarized in Table 1.

## Discussion

In this study, we show that the intra-NTS application of SO_2_ produces dose-dependent hypotension and bradycardia in anesthetized rats, which are most likely mediated by activation of the NO/cyclic GMP (cGMP) signal transduction pathway and/or glutamate receptors. Furthermore, bilateral injection of SO_2_ into the NTS was found to significantly reduce BP and HR and attenuate ABR at the NTS level.

SO_2_ has been traditionally viewed as a toxic gas and a serious environmental pollutant. It is, however, also produced endogenously from the metabolism of sulfur-containing amino acids in mammals [13]. The cardiovascular effects of SO_2_ have been extensively studied and include antihypertension, vasodilation, amelioration of vascular remodeling, antioxidative capacities, regulation of lipid metabolism, and intracellular signal transduction [14]. A previous study has shown that SO_2_ and the key enzyme, AAT-1, are distributed widely in the CNS [15]. As a small gas molecule, SO_2_ can also pass into the CNS readily through the blood–brain barrier. The present study was designed to test the hypothesis that SO_2_ influences cardiovascular function by a central mechanism and to investigate the details of this mechanism.

As the first projection site of afferent fibers from arterial baroreceptors and chemoreceptors, the NTS is known to be important in maintaining cardiovascular autonomic and visceral stability [10]. The mechanisms, however, involved in SO_2_-mediated cardiovascular effects in the NTS remain unclear. The present study aimed to explore these mechanisms.

Here, we showed that microinjection of SO_2_ into the NTS produces dose-dependent cardiovascular inhibitory effects, similar to those seen after microinjection of the excitatory amino acid, l-glutamate. l-glutamate is the primary neurotransmitter released from sensory afferents, and its microinjection into the NTS of anesthetized rats reduces arterial pressure [16]. We hypothesized that the cardiovascular effects caused by intra-NTS SO_2_ are mediated by glutamate receptors. We found, however, that the cardiovascular effects of intra-NTS SO_2_ are only partly decreased by pretreatment with KYN. Therefore, these effects may be mediated by alternative mechanisms. Activation of glutamate receptors within the NTS has been shown to enhance ABR in rats [17]. We found that bilateral microinjection of SO_2_ into the NTS inhibited ABR, which indirectly indicates that the cardiovascular effects of intra-NTS SO_2_ are not completely mediated by glutamate receptors.

SO_2_ has been shown to produce dose-dependent vasorelaxing effects in the peripheral cardiovascular system. At low SO_2_ concentration (<450 µmol/l), vasorelaxation involves the big-conductance calcium ion (Ca^2+^)-activated K^+^ (BKCa) channel, whereas at high SO_2_ concentration (>500 µmol/l), it is associated with K_ATP_ channel activation and the L-type calcium channel (L-Ca^2+^) channel [18]. Furthermore, previous studies using SO_2_ derivatives have shown that the relaxing effect of SO_2_ is related to the Prostacyclin-adenylyl cyclase-cyclic adenosine 3’,5’-monophosphate protein-kinase A (PGI_2_-AC-cAMP-PKA) signaling pathway [19]. Hence, ion channels, such as L-Ca^2+^, K_ATP_, and BKCa channels, as well as cGMP and cAMP pathways, play important roles in the effects of SO_2_ on vasodilation. Pharmacological studies have shown that K_ATP_ channels, l-type calcium channels, and NO are widely distributed in the NTS [20,21]. These reports led us to speculate that the cardiovascular functions of intra-NTS SO_2_ may be mediated by K_ATP_ channels, l-type calcium channels, or NO. To test this hypothesis, we microinjected the K_ATP_ channel blocker, glibenclamide, the l-type calcium channel blocker, nicardipine, or the nonselective inhibitor of NO synthetases, l-NAME, prior to microinjection of SO_2_. Neither glibenclamide nor nicardipine significantly influenced the cardiovascular effects of intra-NTS SO_2_. This suggests that the mechanism whereby intra-NTS SO_2_ induced hypotension and bradycardia did not involve K_ATP_ channels or l-type calcium channels. Pretreatment with l-NAME, however, significantly decreased the hypotension and bradycardia induced by intra-NTS injection of SO_2_. Furthermore, the NO-sensitive guanylyl cyclase inhibitor, ODQ, almost abolished the hypotension and bradycardia induced by intra-NTS injection of SO_2_. It has been reported that SO_2_-induced relaxation of phenylephrine-precontracted rat aortic rings is mediated by endothelial nitric oxide synthase [22]. Our present study also suggested that the hypotension and bradycardia induced by intra-NTS SO_2_ are mediated by the activation of the NO/cGMP signal transduction pathway and/or glutamate receptors.

## Acknowledgements

This work was supported by National Natural Science Foundation of China (No. 30700266), Natural Science Foundation of Gansu province of China (No. 17JR5RA263), Science and Technology Program of Lanzhou, China (No. 2015-2-65), and Medical Scientific Research Foundation of Gansu Province of China (No. GSWSKY2016-20).

## Conflicts of interest

There are no conflicts of interest.

## References

[1] Haider SS Effects of exhaust pollutant sulfur dioxide on lipid metabolism of guinea pig organs. Ind Health. 1985; 23:81–87 4055439 10.2486/indhealth.23.81

[2] Yorifuji T Kashima S Suryadhi MaH Abudureyimu K Acute exposure to sulfur dioxide and mortality: historical data from Yokkaichi, Japan. Arch Environ Occup Health. 2018 1–8. [Epub ahead of print] 10.1080/19338244.2018.1434474 29384437

[3] Huang Y Tang C Du J Jin H Endogenous sulfur dioxide: a new member of gasotransmitter family in the cardiovascular system. Oxid Med Cell Longev. 2016; 2016:8961951 26839635 10.1155/2016/8961951 PMC4709694

[4] Chen SS Tang CS Jin HF DU JB Sulfur dioxide acts as a novel endogenous gaseous signaling molecule in the cardiovascular system. Chin Med J (Engl). 2011; 124:1901–1905 21740851

[5] Li B Lu Y Yue JY Cai HY Zhang Y Chai C Cao N The distribution of endogenous sulfur dioxide in septic rats. Journal of Medical Research. 2013; 42:31–34

[6] Li B Chai C Lu Y Zhang Y Cao N Effects of sulfur dioxide on blood pressure and heart rate in anesthetized rats in the ventrolateral medulla of the medulla. Chinese Journal of Hypertension. 2013; 21:272–278

[7] Colombari E Sato MA Cravo SL Bergamaschi CT Campos RR Jr Lopes OU Role of the medulla oblongata in hypertension. Hypertension. 2001; 38:549–554 11566929 10.1161/01.hyp.38.3.549

[8] Cravo SL Campos RR Colombari E Sato MA Bergamaschi CM Pedrino GR Role of the medulla oblongata in normal and high arterial blood pressure regulation: the contribution of escola paulista de medicina - UNIFESP. An Acad Bras Cienc. 2009; 81:589–603 19722026 10.1590/s0001-37652009000300021

[9] Ang R Abramowitz J Birnbaumer L Gourine AV Tinker A The role of GalphaO-mediated signaling in the rostral ventrolateral medulla oblongata in cardiovascular reflexes and control of cardiac ventricular excitability. Physiol Rep. 2016; 4:e12860 27528004 10.14814/phy2.12860 PMC4985541

[10] Guyenet PG The sympathetic control of blood pressure. Nat Rev Neurosci. 2006; 7:335–346 16760914 10.1038/nrn1902

[11] Ondicova K Mravec B Multilevel interactions between the sympathetic and parasympathetic nervous systems: a minireview. Endocr Regul. 2010; 44:69–75 20429636 10.4149/endo_2010_02_69

[12] Lu Y Wu YS Chen DS Wang MM Wang WZ Yuan WJ Microinjection of salusin-beta into the nucleus tractus solitarii inhibits cardiovascular function by suppressing presympathetic neurons in rostral ventrolateral medulla in rats. Physiol Res. 2015; 64:161–171 25317687 10.33549/physiolres.932616

[13] Jin HF Du SX Zhao X Zhang SQ Tian Y Bu DF [Significance of endogenous sulfur dioxide in the regulation of cardiovascular system]. Beijing Da Xue Xue Bao Yi Xue Ban. 2007; 39:423–425 17657274

[14] Tian H Advances in the study on endogenous sulfur dioxide in the cardiovascular system. Chin Med J (Engl). 2014; 127:3803–3807 25382339

[15] Yu W Liu D Liang C Ochs T Chen S Chen S Sulfur dioxide protects against collagen accumulation in pulmonary artery in association with downregulation of the transforming growth factor beta1/smad pathway in pulmonary hypertensive rats. J Am Heart Assoc. 2016; 5:e003910 27792648 10.1161/JAHA.116.003910 PMC5121494

[16] Granato ÁS Gomes PM Martins Sá RW Borges GS Alzamora AC de Oliveira LB Cardiovascular responses to l-glutamate microinjection into the NTS are abrogated by reduced glutathione. Neurosci Lett. 2017; 642:142–147 28189741 10.1016/j.neulet.2017.02.019 PMC5810122

[17] Talman WT Perrone MH Reis DJ Acute hypertension after the local injection of kainic acid into the nucleus tractus solitarii of rats. Circ Res. 1981; 48:292–298 7460203 10.1161/01.res.48.2.292

[18] Li J Meng Z The role of sulfur dioxide as an endogenous gaseous vasoactive factor in synergy with nitric oxide. Nitric Oxide. 2009; 20:166–174 19135162 10.1016/j.niox.2008.12.003

[19] Liu D Jin H Tang C Du J Sulfur dioxide: a novel gaseous signal in the regulation of cardiovascular functions. Mini Rev Med Chem. 2010; 10:1039–1045 20540708 10.2174/1389557511009011039

[20] Dallaporta M Perrin J Orsini JC Involvement of adenosine triphosphate-sensitive K+ channels in glucose-sensing in the rat solitary tract nucleus. Neurosci Lett. 2000; 278:77–80 10643805 10.1016/s0304-3940(99)00898-8

[21] Rhim H Toth PT Miller RJ Mechanism of inhibition of calcium channels in rat nucleus tractus solitarius by neurotransmitters. Br J Pharmacol. 1996; 118:1341–1350 8832055 10.1111/j.1476-5381.1996.tb15543.x PMC1909685

[22] Yao Q Huang Y Liu AD Zhu M Liu J Yan H The vasodilatory effect of sulfur dioxide via SGC/cGMP/PKG pathway in association with sulfhydryl-dependent dimerization. Am J Physiol Regul Integr Comp Physiol. 2016; 310:R1073–R1080 27009048 10.1152/ajpregu.00101.2015

